# Biomass and Species Diversity of Different Alpine Plant Communities Respond Differently to Nitrogen Deposition and Experimental Warming

**DOI:** 10.3390/plants10122719

**Published:** 2021-12-10

**Authors:** Emmanuella A. Kwaku, Shikui Dong, Hao Shen, Wei Li, Wei Sha, Xukun Su, Yong Zhang, Shuai Li, Xiaoxia Gao, Shiliang Liu, Jianbin Shi, Xiaowen Li, Quanru Liu, Zhenzhen Zhao

**Affiliations:** 1 School of Grassland Science, Beijing Forestry University, Beijing 100083, China; emmkwaku.17@gmail.com; 2 State Key Joint Laboratory of Environmental Sanitation and Pollution Control, School of Environment, Beijing Normal University, Beijing 100875, China; weili@bnu.edu.cn (W.L.); weisha@pku.edu.cn (W.S.); shuaili@sxau.edu.cn (S.L.); gaoxiaoxia0113@163.com (X.G.); shiliangliu@bnu.edu.cn (S.L.); jbshi@bnu.edu.cn (J.S.); xwli_bnu@163.com (X.L.); 3 Research Center for Eco-Environment, Chinese Academy of Sciences, Beijing 100085, China; suxukun1985@163.com; 4 National Plateau Wetland Research Center, College of Wetlands, Southwest Forestry University, Kunming 650224, China; zhy1902@126.com; 5 School of Life Sciences, Beijing Normal University, Beijing 100875, China; liuquanru@bnu.edu.cn; 6 School of Environmental and Chemical Engineering, Shanghai University, Shanghai 200444, China

**Keywords:** nitrogen addition, warming, diversity, productivity, alpine grasslands

## Abstract

The ability of fragile ecosystems of alpine regions to adapt and thrive under warming and nitrogen deposition is a pressing conservation concern. The lack of information on how these ecosystems respond to the combined impacts of elevated levels of nitrogen and a warming climate limits the sustainable management approaches of alpine grasslands. In this study, we experimented using a completely random blocked design to examine the effects of warming and nitrogen deposition on the aboveground biomass and diversity of alpine grassland plant communities. The experiment was carried out from 2015 to 2018 in four vegetation types, e.g., alpine desert, alpine desert steppe, alpine marsh, and alpine salinised meadow, in the Aerjin Mountain Nature Reserve (AMNR) on the Qinghai–Tibetan Plateau (QTP). We found that W (warming) and WN (warming plus N deposition) treatment significantly increased the aboveground biomass of all the vegetation types (*p* < 0.05) in 2018. However, W and WN treatment only significantly increased the Shannon diversity of salinised meadows in 2018 and had no significant effect on the Shannon diversity of other vegetation types. Such results suggested that long-term nitrogen deposition and warming can consistently stimulate biomass accumulation of the alpine plant communities. Compared with other vegetation types, the diversity of alpine salinised meadows are generally more susceptible to long-term warming and warming combined with N deposition. Warming accounts many of such variabilities, while short-term N deposition alone may not significantly have an evident effect on the productivity and diversity of alpine grasslands. Our findings suggested that the effects of short-term (≤4 years) N deposition on alpine vegetation productivity and diversity were minimal, while long-term warming (>4 years) will be much more favourable for alpine vegetation.

## 1. Introduction

The global surface temperature is expected to increase by about 4 °C by 2100 [1]. Parallel to these warming trends is the dramatic increase in anthropogenic nitrogen emission and deposition on a global scale [2]. This can influence critical ecological processes, including range and structural shifts in plant species [3]. Under growing effects of global environmental changes, it is important to understand the response of vegetation to the combined effects of warming and nitrogen deposition.

Previous studies have found that temperature can modify nitrogen mineralisation [4,5], which is often exacerbated by changes in land use [6,7,8]. Some studies found that the number of vascular plants generally tends to increase with the increase in temperature and nitrogen deposition; this may be because temperature and nutrients are the main limiting factors in alpine ecosystems [9]. However, plant community and plant species might respond differently to nitrogen deposition due to variations in nitrogen use efficiency or their differential adaptability [10,11]. The general findings of previous studies indicated a decline in species richness in various ecosystems such as acidic, saline mountain and temperate grasslands [12,13,14]. The results regarding the effects of increased nitrogen deposition on plants biomass and diversity vary; positive, neutral and negative findings have been reported [15,16,17,18]. Plant species richness is an important indicator for diversity, and a previous study found that species richness might decline as a result of increased nitrogen deposition [19]. N deposition has the potential to decrease plant richness by favouring the plant species that can better adapt to high nutrient loads [19]. Under high N deposition, numbers of nitrophilous plant species may increase, while numbers of N-sensitive plant species may decrease. Additionally, N deposition can cause soil acidification, which will impact plant growth and thus cause species loss.

Warming and nitrogen deposition have impacted ecosystem multi-functionalities and contributed to the global carbon budget [20]. These synergistic effects bring big challenges to the grassland conservation domain [21,22]; previous experiments have demonstrated warming and nitrogen deposition stimulate plants biomass and enhance the photosynthetic abilities of key plants [23,24,25]. The effects of elevated nitrogen deposition on plants can also be distinguishable by drier months and at the species level [23]. Warming and nitrogen deposition significantly increased plant community composition, diversity and richness due to long-term increases in temperature and nitrogen deposition levels [11]. Previous results about warming effects on plant biomass are not consistent, because positive [26], negative [27] and neutral [28] results were found. So far, the extent to which alpine vegetation responds and serves as a sink for nitrogen under climate (temperature) variability is poorly studied. Understanding further how alpine vegetation responds to nitrogen deposition and increased temperature is essential for conservation efforts for fragile alpine ecosystems.

The QTP is considered the “third pole” and a key alpine biome in the world [29,30,31]. The alpine vegetation on the QTP is highly sensitive to nitrogen deposition and warming [32,33,34]. Several key experiments have been performed across the QTP to quantify the extent of warming and nitrogen influences on alpine plant productivity [35,36,37,38,39]. However, most investigations regarding warming and nitrogen deposition did not account for how different vegetation types respond to warming and N deposition at the same time and sites. Here, we assess the synergistic effects of simulated warming and nitrogen deposition on plant productivity and diversity across different vegetation types and over 4 years to inform global environmental change policies. The operating hypotheses for this study are that: (1) the duration of warming and nitrogen addition would influence plant species diversity and aboveground biomass and (2) the effect of warming and nitrogen addition differs across different vegetation types, including alpine desert, alpine desert steppe, alpine marsh and alpine salinised meadow. The sensitivity of plants to the combined effects of warming and nitrogen is important for devising sustainable conservation management efforts and enhances our understanding of responses of alpine vegetation to global environmental changes.

## 2. Materials and Methods

### 2.1. Study Sites and Experimental Treatment

The study was conducted in the Aerjin Mountain Nature Reserve (AMNR), which is located in the northwestern QTP at 87°10′18″–91° E, and 36°00′49″–37° N. The QTP has a total land size of 2.6 million km^2^ and is on average 4000 m above mean sea level. It accounts for over 50–60% of the total carbon budget from grassland ecosystems in China [40]. The plateau has abundant and diverse species, making it one of the biodiversity conservation hotspots in China [41]. As a sustainable future is a goal, several tracts of the QTP are protected, including Aerjin Mountain, Changtang and the Hohxil National Nature Reserve. The Aerjin Mountain Nature Reserve (AMNR) has a land size of 45,000 km^2^ and it is surrounded by high mountains. The reserve has no frost-free periods. The climate is cold, dry and windy. The average temperature at the QTP has increased by about 0.3 °C per decade over the last 50 years, a rate that is faster compared to the average temperature of China [42], with an annual mean temperature below zero degrees and an average annual rainfall of about 300 mm. According to the International Soil Classification system, the dominant soils in the AMNR are loam and loam–sandy. The major vegetation types in the AMNR are desert, desert steppe, marsh, salinised meadow and sparse alpine vegetation. The study was conducted on four different vegetation types. *Salsola abrotanoides* Bunge is the dominating species in the desert located in the southeastern AMNR, desert steppe is dominated by S*tipa purpurea* Griseb found in the southwestern AMNR, marsh is dominated by *Kobresia robusta* Maximowicz and *Carex moorcroftii* Falc. Ex Boott located around the rivers and lakes and *Allium polyrhizum* Turcz. ex Regel and *Kalidium foliatum* (Pall.) Moq. dominate in the salinised meadow around the edges of the south and north of the AMNR.

### 2.2. Experimental Design

**Figure 1:**
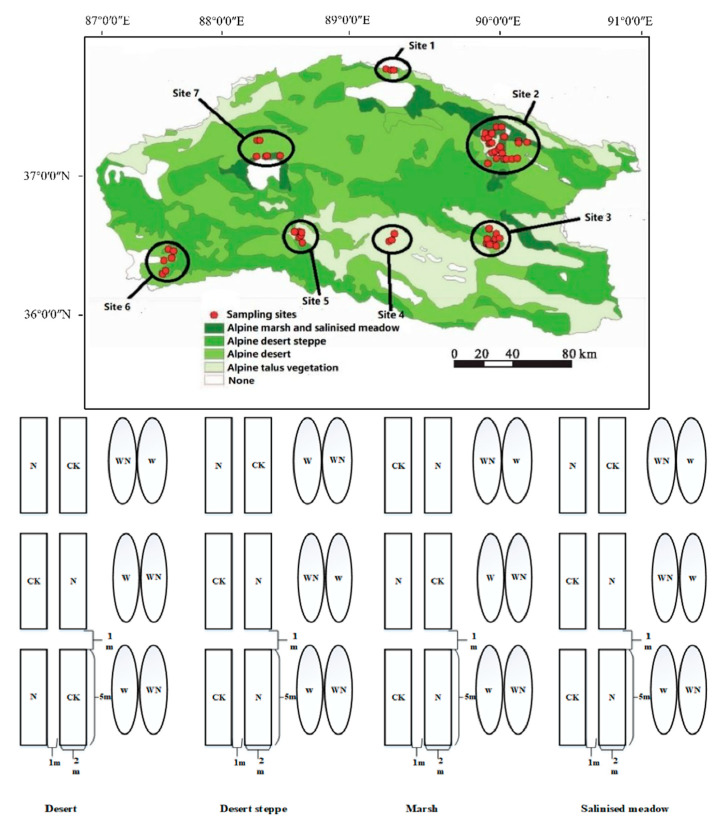
Main vegetation types, sampling sites and treatment in Aerjin Mountain Nature Reserve. The experimental plots: CK = no N addition, N = 16 kg N ha^−1^ year^−1^, W = warming and WN = warming + N addition. The boxes and circles in this figure represent the shape of plots.

To explore responses of alpine plant communities to experimental warming and nitrogen addition, we carried out complete randomised block experiments across four distinct vegetation types (hereafter “sites”), including marsh, salinised meadow, desert and desert steppe. For each type of vegetation, 12 plots (replicates) of 2 m × 5 m were randomly placed. The plots were set up for four treatments: a control treatment without nitrogen and warming (CK), a warming treatment (W), a warming and nitrogen treatment (WN) and a nitrogen treatment (N). For the nitrogen addition treatment, a single level of ammonium nitrate (NH_4_NO_3_) was applied (Figure 1). Given that the nitrogen deposition on the QTP is 8 kg N ha^−1^ year^−1^ as reported by Lü and Tian (2007) [29], we set the N to 16 kg N ha^−1^ year^−1^ to represent two times the annual N deposition on the QTP.

In the warming plot (W), the temperature was elevated by approximately 1.8–2.2 °C more than that of the control plot. During treatment application, three open-top chambers (OTCs, Figure S1) were placed randomly on W subplots. OTCs permit the quasi-natural transmittance of visible wavelengths and reduce the transmittance of reradiated infrared wavelengths [43]. The OTCs used in this study were comparable to the hexagonal chambers designed by Marion et al. (1997) [43] and consisted of transparent polycarbonate (50 cm high, 1.7 m at the top and 2.4 m at the base) [44]. The OTCs were elevated 10 cm above the soil surface to allow airflow [45]. The installation of OTCs in the field was carried out by adhering to the standard procedures of the International Tundra Experiment (ITEX) [46]. Soil temperature sensors (5TM; Decagon Devices, Pullman, WA, USA) were mounted at 5 cm depth in all the plots. The 5TM sensors (CR1000, Campbell Scientific; Logan, Utah; USA) recorded the temperature at 5 s intervals, after which an average value of 2 min was recorded [44]. The experiments were repeated for the 192 subplots (4 treatments × 3 replicates × 4 grassland types × 4 years). The experiments were carried out at the peak of the growing season in late July to late August of 2015 to 2018.

### 2.3. Plant Productivity and Diversity Calculation

The percentage of species, cover and richness were estimated by field visual observation according to Ren’s methods (1998) [47]. We randomly placed one 1 m × 1 m quadrat for the plant species composition survey, and species names, the number of species, plant heights and plant coverages were all recorded. During the peak growth season (July–August) in each year, the aboveground plant parts in each plot were harvested after investigation and dried at 70 °C in the lab for the measurement of aboveground biomass. Species richness was calculated as the total number of plants species in the plot. The relative abundance of each species was calculated as the ratio between individual species and the different treatment levels and the total number of species in the plots [47]. Shannon–Weiner’s diversity index was used to quantify species diversity status in all the habitats [48]. The index assumes that individuals are randomly sampled from an ‘infinitely large’ population and all the species from a community are included in the sample. Species evenness relates to how abundantly the species occurs in the plant community, and it is related inversely to species dominance.

### 2.4. Statistical Analysis

Statistical analysis was performed with R 3.6.1. A two-way analysis of variance (ANOVA) was used to test the effects of the year, treatment and the interactions between year and treatment on the plant biomass, cover, height, richness, and diversity. The figure that displays the response of total community aboveground biomass and Shannon diversity to nitrogen addition and experimental warming in the desert (DST), desert steppe (DSP), marsh (MSH) and salinised meadow (SLM) from 2015 to 2018 was produced by Origin Pro 8.0 (OriginLab Corporation; Northampton, MA, USA).

## 3. Results

### 3.1. Effects of N, W and WN on Aboveground Plant Biomass and Plant Diversity

**Figure 2:**
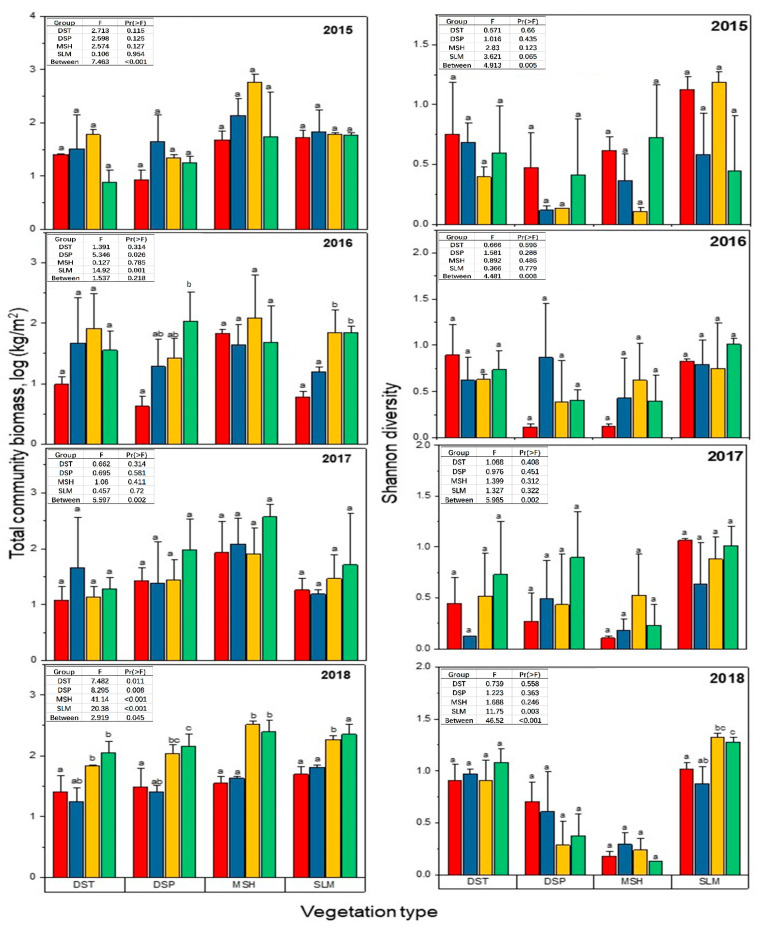
Response of total community aboveground biomass (Kg/m^2^) and Shannon diversity to nitrogen addition and experimental warming in desert (DST), desert steppe (DSP), marsh (MSH) and salinised meadow from 2015 to 2018. Treatments (within year) with the same letter are not significantly different. The different colour in the graph represents the experimental treatment, the red colour = CK, blue colour = N, yellow colour = W and green colour = WN.

The experimental treatments had varying effects on the aboveground plant biomass and plant diversity across vegetation types and sampling years (Figure 2). No significant differences of aboveground plant biomass were observed among different experimental treatments for all vegetation types in 2015 (*p* > 0.05) and 2017 (*p* > 0.05). In 2016, the aboveground biomass of the alpine desert steppe was significantly higher for WN compared to CK (*p* < 0.05). Compared with CK, the aboveground biomass was significantly higher in the WN and N treatments in the alpine salinised meadow (*p* < 0.05). In 2018, the aboveground biomass was significantly higher in the W and WN treatments (*p* < 0.05) compared with CK in all the vegetation types. Significant differences in plant species diversity were not detected among the different experimental treatments for all vegetation types in 2015 (*p* < 0.05), 2016 (*p* < 0.05) and 2017 (*p* < 0.05). In 2018, the W and WN treatments significantly increased the Shannon diversity of the salinised meadow (*p* < 0.05).

### 3.2. Interactions between Experimental Treatment and Sampling Year on Plant Community

As shown in Table 1, the interaction between the sampling year and experimental treatment were observed regarding the coverage (*p* < 0.001) of the alpine desert. Sampling year alone had a significant effect on coverage (*p* < 0.05), height (*p* < 0.001), richness (*p* < 0.01), Shannon (*p* < 0.01) and Pielou indies (*p* < 0.001). Treatment alone only had a significant effect on coverage (*p* < 0.01) and height (*p* < 0.05).

In the alpine marsh, interaction between the sampling year and experimental treatment was observed on the height (*p* < 0.01). Sampling year alone only had a significant effect on coverage (*p* < 0.01). Treatment alone only had a significant effect on biomass (*p* < 0.01) and coverage (*p* < 0.05).

In the alpine desert steppe, the interaction between the sampling year and experimental treatment were only observed regarding the coverage (*p* < 0.05). Sampling year alone had a significant effect on biomass (*p* < 0.05), coverage (*p* < 0.001) and richness (*p* < 0.01). Treatment alone only had a significant effect on biomass (*p* < 0.01) and coverage (*p* < 0.01).

In the salinised meadow, interaction between the sampling year and experimental treatment were not observed. Sampling year alone had a significant effect on biomass (*p* < 0.001), coverage (*p* < 0.05) and richness (*p* < 0.001). Treatment alone only had a significant effect on biomass (*p* < 0.01) and Shannon index (*p* < 0.05).

### 3.3. Relationship among Plant Biomass, Coverage, Evenness, Height, Richness and Diversity

Table 2 shows Pearson’s Moment correlations for the community cover, aboveground plant biomass, species evenness, plant height, richness, Shannon diversity and Pielou index. Plant coverage showed a strong correlation with plant biomass (*r* = 0.45; *p* < 0.05). Plant height had a strong correlation with aboveground biomass and coverage (*r* = 0.41, *p* < 0.05; *r* = 0.65, *p* < 0.05, respectively). There was a strong correlation between plant richness and aboveground biomass (*r* = 0.31; *p* < 0.05), coverage (*r* = 0.59; *p* < 0.05), and plant height (*r* = 0.43; *p* < 0.05). There was no strong correlation between plant biomass with Shannon and Pielou indices. Shannon diversity positively correlated with plant height (*r* = 0.27; *p* < 0.05). There was a strong positive correlation between Pielou evenness and height (*r* = 0.27; *p* < 0.05) and Shannon diversity (*r* = 0.90; *p* < 0.05). Pielou evenness has a strong association with richness (*r* = −0.19; *p* < 0.05) (Table 2).

## 4. Discussion

The responses of plant biomass and diversity to the changes occurring in an environment reflects the resilience of the plant community [34,49]. Our results showed that plant communities (alpine desert, alpine desert steppe, alpine marsh, and alpine salinised meadow) might respond differently to long-term warming and N deposition.

In this study, we observed that warming and warming combined with N deposition treatments stimulated the aboveground biomass of all vegetation types after the four-year-long experiment (2015–2018). This suggested that long-term warming and warming combined with N deposition will increase vegetation productivity in alpine regions. This may be because alpine plant communities are usually limited by cold weather; thus, warming can favour the growth of plants in cold alpine regions. We also found that aboveground biomass increased more under the warming combined with N deposition treatment in the alpine salinised meadow, suggesting that N might still be a limiting factor for plant productivity in alpine salinised meadows. In the alpine desert, alpine desert steppe and alpine marsh, the positive effects of warming on plant biomass were more obvious, while the effects of N deposition were not significant, suggesting that temperature might be an important limiting factor in other vegetation types. Responses of plant communities to warming and N deposition usually vary with vegetation types [50]. Changes in aboveground plant biomass are central in understanding how nitrogen deposition (N) and warming (W) affect the productivity of alpine communities. In the present study, WN and N treatment only obviously increased the aboveground plant biomass of all vegetation types in 2018, suggesting that such effects might need a longer time to be evident. The variation differed across different vegetation types, indicating that the alpine plant communities’ differential responses might be associated with the divergent adaptabilities of different alpine vegetation types to environmental changes [11,12,50].

In this present study, we found that nitrogen deposition alone rarely increased aboveground biomass. This finding is consistent with the results found in the observation of the Wasatch Plateau by Gill (2014) [51]. In contrast, N deposition significantly increased plant aboveground biomass in a temperate old field [3]. The highest increase in plant biomass in the alpine salinised meadow was due to the combined effects of experimental warming and nitrogen deposition. This is consistent with a study that was carried out in the temperate grassland of Songnen Plain in northern China [11]. Though plant diversity decline might be attributed to warming in numerous experiments [52,53,54,55], our studies found that only the Shannon index of the alpine salinised meadow was increased under warming and warming combined with N deposition treatment after the four-year-long experiment. This suggested that the diversity of alpine desert, alpine desert steppe and alpine marsh is much more stable and may not be very susceptible to the influence of short-term (four years) warming and N depositionl other environmental factors, such as climatic variability (precipitation in particular), might indirectly affect co-existing species by altering the composition of the plant community [56]. However, in 2018, warming and warming combined with N deposition significantly increased the plant diversity of the alpine salinised meadow, indicating that the plant diversity of the alpine salinised meadow was more sensitive to short-term (four years) warming and warming combined with N deposition. This finding showed that N deposition alone had no obvious impacts on the species diversity of the alpine plant communities; large disparities might take a longer period (>4 years) to become apparent [11,57].

Generally, there is a positive relationship between plant biomass and coverage [58,59,60,61], and this is consistent with our results. Our results also showed a strong positive relationship between biomass and plant cover. It has been reported that plant productivity in the alpine ecosystem is sensitive to environmental changes, especially temperature [62,63]. Our findings indicate a strong relationship between species diversity and plant productivity variables, which could be due to the unique characteristics of the grasslands.

## 5. Conclusions

Responses of the alpine plant communities varied with different vegetation types to warming, nitrogen deposition and warming plus nitrogen deposition over four years in the well-protected alpine area of the AMNR on the QTP. Long-term (>4 years) warming and warming plus N deposition will significantly increase the productivity of alpine desert, alpine desert steppe, alpine marsh, and alpine salinised meadow. Long-term (>4 years) warming and warming plus N deposition will significantly increase the plant community diversity of alpine salinised meadow. Short-term (≤4 years) N deposition might have no significant effect on plant community productivity and diversity. No obvious interactions were found between warming and N deposition in the short term. Large disparities might take a longer period (>4 years) to become apparent. Warming was shown to be essential in increasing the productivity of alpine vegetation, as the effect of the N deposition was minimal. These findings can further improve our understanding of how alpine vegetation reacts to changes in the environment and help us to have a better sustainable conservation management of alpine grassland in the future.

## Figures and Tables

**Table 1:** Two-way ANOVA for treatment (TRT) and year (YR) and their interactions on the measured variables for four vegetation types.

Factors	Desert						Marsh					
	Biomass	Coverage	Height	Richness	Shannon	Pielou	Biomass	Coverage	Height	Richness	Shannon	Pielou
**Year**	1.303	3.646 *	7.118 ***	5.376 **	5.566 **	8.747 ***	1.278	5.589 **	1.609	2.102	1.943	1.850
**TRT**	1.966	5.228 **	2.935 *	0.398	1.086	1.781	5.151 **	4.045 *	0.311	1.309	0.469	0.706
**Year × TRT**	1.687	4.504 ***	1.843	1.807	0.737	0.695	1.680	1.141	3.903 **	1.539	1.868	1.420
**Factors**	**Desert steppe**						**Salinised meadow**					
	**Biomass**	**Coverage**	**Height**	**Richness**	**Shannon**	**Pielou**	**Biomass**	**Coverage**	**Height**	**Richness**	**Shannon**	**Pielou**
**Year**	3.445 *	7.441 ***	1.107	5.576 **	0.918	2.204 ^+^	8.827 ***	3.808 *	2.711 ^+^	15.317 ***	2.719	2.751 ^+^
**TRT**	6.583 **	5.985 **	1.401	0.580	0.885	1.016	6.786 **	0.396	1.782	1.936	3.056 *	2.684 ^+^
**Year × TRT**	1.588	2.997 *	0.859	1.967 ^+^	1.314	0.887	1.261	0.908	0.459	1.851	1.951	1.973 ^+^

Notes: *p* < 0.001 = ‘***’, *p* < 0.01 = ‘**’, *p* < 0.05 = ‘*’, *p* < 0.1 = ‘+’. Biomass, coverage, height and richness were log-transformed to stabilise the variance before ANOVA was applied.

**Table 2:** Person’s correlation test among plant community properties.

Variables	Biomass	Coverage	Height	Richness	Shannon	Pielou
**Biomass**	1.00					
**Coverage**	0.45 **	1.00				
**Height**	0.41 **	0.65 **	1.00			
**Richness**	0.31 **	0.59 **	0.43 **	1.00		
**Shannon**	−0.12	−0.07	0.27 **	−0.13	1.00	
**Pielou**	−0.13	−0.10	0.27 **	−0.19 **	0.90 **	1.00

Note: **: *p* < 0.05. Biomass, coverage, height and richness were log-transformed.

## Data Availability

All the data supporting this article were included in the main text.

## Supplementary Materials

The following are available online at https://www.mdpi.com/article/10.3390/plants10122719/s1, Figure S1: Truncated cone-shaped open top chamber schematic diagram.
