# Supplementary figures and images for: Biomass and Species Diversity of Different Alpine Plant Communities Respond Differently to Nitrogen Deposition and Experimental Warming
Source: Plants (Basel). 2021 Dec 10;10(12):2719. doi: 10.3390/plants10122719 (PMC8703334; doi:10.3390/plants10122719)

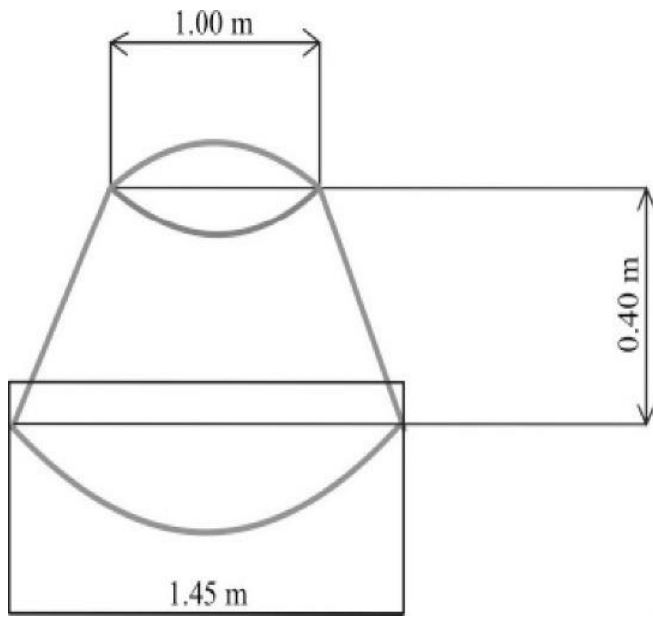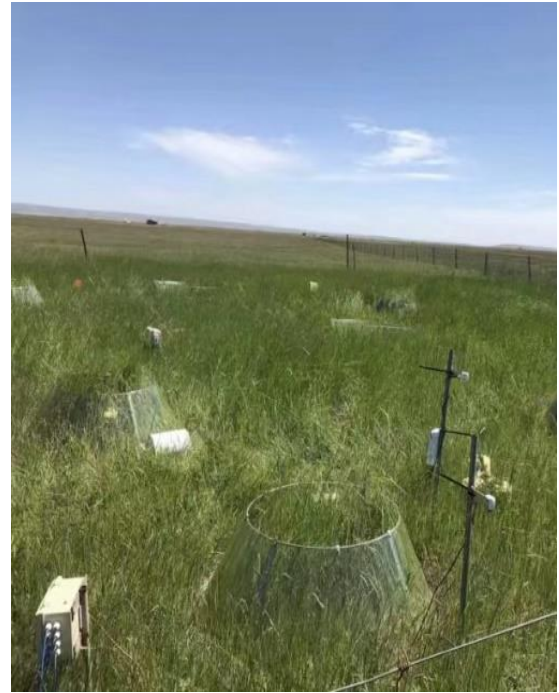

**Figure S1.** Truncated cone-shaped open top chamber schematic diagram.

Supplement: Supplementary file 1 [file plants-10-02719-s001.zip › plants-1430756-supplementary.pdf]
